# Treatment Burden and Uncertainty in the Context of Advanced Multimorbidity: A Focussed Ethnography

**DOI:** 10.1177/10497323251320836

**Published:** 2025-03-14

**Authors:** Chris McParland, Bridget Johnston, Mark Cooper

**Affiliations:** 1 12194 University of Glasgow, School of Medicine, Dentistry and Nursing, Glasgow, UK; 2 3529 NHS Greater Glasgow and Clyde, Glasgow, UK

**Keywords:** multimorbidity, treatment burden, uncertainty, chronic illness, users’ experiences of healthcare

## Abstract

Advanced multimorbidity is the term used to describe when someone has multiple chronic conditions including those which are associated with palliative care needs. People living with advanced multimorbidity have to coordinate and undertake lots of different tasks related to their chronic conditions, and this can lead to them feeling overburdened, and disengagement from treatment regimes. In this study, we sought to explore how this “treatment burden” was experienced by people with advanced multimorbidity and their caregivers. We adopted a focussed ethnographic approach, in which patient/carer dyads (six groups of two, recruited from an emergency department) took part in two semi-structured interviews and kept a participant-led journal of treatment burden experiences. We also offered to observe any burdensome activities, although only one such session was conducted. A reflexive thematic analysis of the data was conducted by a single researcher, in which data were coded both inductively and through the lens of Burden of Treatment Theory, plus two theories of uncertainty (Total Uncertainty and Uncertainty Tolerance). The types of patient work were split between practical tasks (such as taking medicines or going to hospital) and cognitively burdensome activities (such as symptom surveillance and planning ahead). Burden of Treatment Theory was useful in understanding how work was distributed between patients and their relational networks. We found that multidimensional uncertainty mediated the balance between workload and capacity, and we propose a conceptual model of this relationship alongside a suggestion for how interventions can be used to manage uncertainty and burden.

## Background

Multimorbidity is the term used to describe having more than one concurrent chronic condition, where no single condition is considered more central than the others (Boyd & Fortin, 2010). In the general population, around one in three people have multimorbidity (Nguyen et al., 2019), and around half of people who have a chronic condition also have multimorbidity. Multimorbidity is associated with premature mortality, increased healthcare use, and increased costs. For the individual, it leads to worsening health, poorer functional status, and potentially burdensome and fragmented treatment regimens (Skou et al., 2022).

The number of people who have multimorbidity which features severe, life-limiting or palliative conditions is also high and is projected to grow significantly in coming years (Finucane et al., 2021). In this group, the burdens of medication management, treatment adherence, and various other forms of patient “work” can become unmanageable (Mason et al., 2016).

The term treatment burden is used to describe the work associated with healthcare and the effects that it has on patients (Eton et al., 2012). It is a dynamic, multidimensional phenomenon which comprises both subjective and objective burdens (Sav et al., 2015). Burden of Treatment Theory was proposed by May et al. (2014) as a structural model which can be used to understand treatment burden at multiple levels. It draws on other theories including Normalisation Process Theory (NPT) (May & Finch, 2009) and the Cumulative Complexity Model (Shippee et al., 2012), and has been used as an analytical lens with a variety of populations across the world (Gallacher et al., 2022). Treatment burden is a core outcome in multimorbidity research (Smith et al., 2018), and there are a variety of validated tools which have been developed to measure treatment burden (Duncan et al., 2018; Eton et al., 2012; Tran et al., 2012, 2014). However, less is known about the qualitative experience of treatment burden for people with advanced multimorbidity and their caregivers.

## Aim of the Study

The aim of the study was to understand how people with advanced multimorbidity and those who care for them experience treatment burden.

## Methods

### Design

The study employed a focussed ethnographic approach which incorporated semi-structured interviews, participant journaling, and optional observation sessions.

Ethnographic research seeks to understand cultures, often through the lens of an “outsider” researcher engaged in prolonged observation of a particular group (Cruz & Higginbottom, 2013; Gullion, 2016).Yet, instances exist where a culture may be geographically or socially disparate, where the members of a culture are linked not by proximity but through shared exposure to cultural stimuli such as having a chronic condition (Morse, 2014). In such cases, a focussed ethnographic approach may be more appropriate.

Differences between a classical ethnographic approach and the focussed design employed here include a pre-specified and focussed aim, short-term engagement with the study population, limited (or no) interaction between participants, and a researcher with a priori knowledge of the phenomena under investigation (Higginbottom et al., 2013; Wall, 2015). In planning the study, we intended to address the aim described above, and understood that the participants were unlikely to have any engagement with one another. We were keen to avoid imposing additional burden through prolonged observation; therefore, we limited the number of direct interactions and utilized participant journals. The project was also informed by existing theories and explored how these represented the experience of those involved.

Aspects of the traditional ethnographic approach which are maintained include the focus on participants as a cultural unit, maintaining a non-judgmental, holistic orientation, and considering both the (emic) perspective of participants as insiders and the (etic) perspective of the researcher as an outsider (Fetterman, 2020). We analyzed the data with a view to understanding the common cultural experience of people with multimorbidity and their carers rather than individual case studies and adopted a holistic (disease-agnostic) approach to considering multimorbidity from the perspective of the individual. We present the emic perspective of the participants through direct quotations and the opportunity to journal their experiences independently. The etic perspective of the researcher as outsider was integrated through the use of a reflexive journal.

### Theoretical Framework

This research adopts a critical realist metatheoretical position, which involves the following assumptions. Firstly, that causal inferences about a shared reality can be made based on empirical observations. Secondly, that epistemic activity involves the generation of theories to explain these observations. And finally, that epistemic relativism allows for multiple theories to coexist (Bhaskar, 2013). The decision to adopt one theoretical explanation over another is based on the extent to which these theories can account for observed events (Bhaskar, 2009).

**Figure 1.**
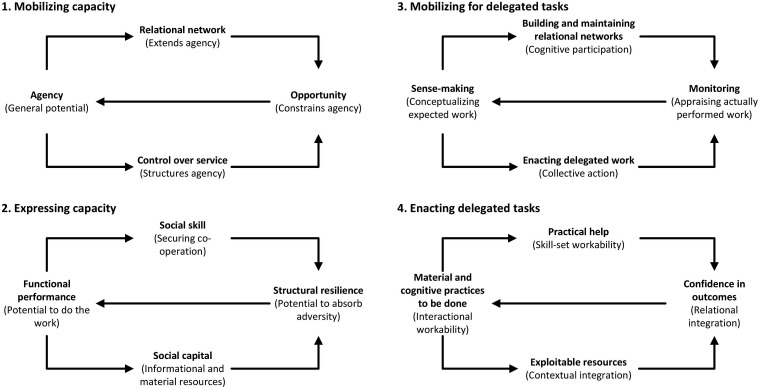
Burden of treatment theory, reproduced from May et al. (2014).

This study is informed by Burden of Treatment Theory (May et al., 2014). Four of the five generative mechanisms described in the original paper are reproduced in Figure 1. The fifth generative mechanism relates to the design of interventions, which will be discussed later. At the simplest level, Burden of Treatment Theory posits that agency (the ability of individual to undertake work) is extended through their relational network (relatives, friends, trusted healthcare professionals), that it is structured through the healthcare services available, and constrained by the opportunities presented within the healthcare service. The capacity to do this work is also mediated by myriad factors, including the ability of network members to enlist co-operation, their access to informational and material resources, the structural resilience of the network, and the ability to conceptualize the work, among others. For a full explanation of the theory, see May et al. (2014).

The theories of Uncertainty Tolerance (Hillen et al., 2017) and Total Uncertainty (Etkind et al., 2022) were also added in response to early findings. Inductive coding led to the development of the theme of “uncertainty,” and these theories provided a further analytical lens. Based on a systematic review of literature involving people with advanced multimorbidity, Etkind et al. (2022) describe total uncertainty as a continually changing multidimensional phenomenon comprising physical uncertainty (about managing multiple illnesses), practical uncertainty (about fragmented care and poor communication), social uncertainty (being unsure of healthcare providers, often due to their perceived inability to meet the needs of patients and carers), and psychological or existential uncertainty (feeling overwhelmed). Hillen et al. (2017) propose a conceptual model of uncertainty tolerance, in which uncertainty (the conscious awareness of one’s own ignorance about something) leads to cognitive, emotional, and behavioral responses, and the extent to which these responses are positive or negative is influenced by external mediators (such as the features of the uncertainty stimulus, the individual, or the situation in which uncertainty is encountered.

### Participants and Recruitment

The units of analysis in this study were patient/carer dyads. Eligible patients had two or more chronic conditions including at least one of the following: cancer, chronic heart failure, chronic kidney disease, chronic obstructive pulmonary disease, or dementia. We selected conditions which are often considered severe or life-limiting, or are associated with palliative care needs, as we were keen to speak with people who had regular interactions with healthcare services rather than conditions which required little management. These conditions were selected as they were some of the most prevalent conditions in the population (McParland et al., 2022). Both participants and carers also had to be aged 18 or older, live in the health board area, and be able to read and speak English (provisions were made to support people with hearing or visual impairments). If patients did not have capacity to consent (for example, due to dementia or other cognitive impairment), then it was possible in some cases to obtain consent from a legal representative, as is permitted in Scottish law (The Scottish Government, 2000). This requires that the project is reviewed by a specific national ethics committee, as was the case for this study. Ensuring people were not excluded from the study due to cognitive impairment was seen to be important, as more than 20% of people with multimorbidity attending the emergency department in this geographic area have a condition which may be associated with cognitive impairment (McParland et al., 2022). The recruitment target was between six and ten patient/carer dyads. This was a pragmatic decision based on what was deemed to be achievable within the recruitment period, what was expected to produce a manageable amount of data given the multimodal data collection approach, and was still adequate to ensure a variety of conditions and circumstances were represented.

The emergency department was selected for recruitment purposes for three reasons. Firstly, the epidemiological work which preceded this study indicated a large proportion of people who may be potentially eligible attended the emergency department. Secondly, it was considered important to access a generalist setting where people would have varied reasons for attendance and diverse ongoing care plans (i.e., admission, discharge, and referral) rather than specialist or inpatient settings. Finally, while there is a paucity of research involving people with multimorbidity in the emergency department, it was assumed that the disruptive nature of an emergency attendance at hospital would provide a useful mechanism to begin exploring the nature of treatment burden.

Clinical teams in the emergency department were made aware of the inclusion/exclusion criteria prior to the start of the study and were able to refer potentially interested people to the researcher. Providing that the patient had assented to being approached, the researcher would introduce themselves, describe the study, perform screening, and provide documentation for them to read. This also required the carer to be present in the emergency department. If they were happy to be contacted at a later date, the researcher called the patient and carer after they had been discharged to confirm if they wanted to take part. If they agreed, arrangements were made to attend the patient’s home in order to obtain informed consent from both them and the carer. All research activities took place after the patient had been discharged from hospital.

In cases where the patient did not have capacity to consent (due to cognitive impairment), the carer would complete a separate consent form designed to be completed by legal representatives on behalf of adults with incapacity. In all cases, patients and carers were made aware of their right to withdraw at any time and without giving a reason.

### Data Collection and Data Analysis

The following types of data were used for analysis: verbatim transcripts of interviews, participant journals, researcher field notes, and reflexive journal entries completed by the researcher.

Participants took part in two semi-structured interviews, usually in their own home (one dyad were interviewed using video-conferencing software for their convenience), at the beginning and end of the study period. The second interview was intended to revisit the concept of treatment burden after participants had the opportunity to journal and reflect on how it affected their lives. Both participants and carers were interviewed together, and the interview was audio recorded and transcribed verbatim. Questions were asked of participants concurrently and discussion was encouraged. Interviews lasted approximately 1 hour; the shortest recording was 37 minutes and the longest was 1 hour and 28 minutes. The date of the second interview was arranged at the convenience of the interviewees, but a period of at least 4 weeks was preferable to ensure adequate time for journaling of activities. The interview topic guide was wide-ranging, and shaped by the literature on treatment burden, issues raised by members of the patient and public involvement group, alongside the domains covered by two validated tools: the Treatment Burden Questionnaire (Tran et al., 2014) and Multimorbidity Treatment Burden Questionnaire (Duncan et al., 2018). The topic guide was flexible enough to allow the conversation to be guided by the participants and for issues raised during the first interview to be revisited during the second. The topic guide is available as an online appendix.

Participants were also provided with notebooks and writing materials (digital audio recorders were available to those who had trouble writing) with which they were advised to document any burdensome experiences they had between the opening and closing interviews. There was no required format, and participants were encouraged to document their experiences in as much or as little detail as they wished. These journals were collected by the researcher at the closing interview.

Participants also had the option to nominate a burdensome activity they would like the researcher to observe; it was anticipated that things like travelling to appointments, organizing medications, or taking exercise would feature. However, most observations of medication management were ad-hoc and took place alongside the interviews, as was an observed exercise session for one participant.

All transcripts were prepared by a professional transcription service and pseudonymized by a member of the research team.

All data items (transcripts, participant journals, notes, and reflexive journals) were either scanned or directly imported into NVivo (QSR International Pty Ltd, 2020) for analysis, and analyzed concurrently. Data were analyzed using reflexive thematic analysis, as outlined by Braun and Clarke (2022). The first author alone conducted data collection and analysis, hence the focus on maintaining a reflexive journal. Familiarization with the dataset was achieved by reading and re-reading transcripts and listening to audio recordings (which also allowed the accuracy of transcripts to be verified). Two concurrent parallel approaches to initial coding were employed; data were coded to an a priori thematic framework based on Burden of Treatment Theory (May et al., 2014), alongside line-by-line inductive coding. Candidate themes were then generated both within the a priori framework and from inductive codes, and these progressed through several iterations of development and review.

Reflexive diaries kept by the researcher were also incorporated into the analysis, and it was through reviewing these and the inductive codes that the concept of “uncertainty” was developed. Further reading around this concept resulted in two theories which were then incorporated into the inductive analytical framework: Uncertainty Tolerance (Hillen et al., 2017) and Total Uncertainty (Etkind et al., 2022). Relationships between the inductive framework (uncertainty) and a priori framework (burden of treatment theory) were then explored to identify relationships between the two theoretical concepts. Refining and defining the final themes resulted in a transitive theoretical model based on the concepts of the theories underpinning the analysis.

### Reflexivity Statement

Recruitment, data collection, analysis, and writing up was conducted by a sole researcher. While this is considered acceptable in reflexive thematic analysis (Braun & Clarke, 2022), it is important to acknowledge the characteristics of the researcher and how they may impact the research (Braun & Clarke, 2024).

The researcher is a white male in his 30s, native to the country in which the research was conducted, and previously worked as a nurse in the emergency department where recruitment took place. At the time of this study, the researcher was a clinical academic PhD student working a proportion of his time as a clinical research nurse.

This familiarity with the population and the environment (from a clinical perspective) meant that the researcher was able to work closely with the clinical team to identify participants who were likely to provide a range of perspectives on treatment burden. However, it also brought assumptions about the types of things which may comprise treatment burden in this cohort (clinical or logistical tasks such as taking medicines and travelling around the health board, or invasive and personal care tasks like stoma care, catheterization, self-administered injections, and blood sugar monitoring). Several steps were taken to avoid being biased toward identification of these tasks. Firstly, a topic guide which covered a variety of subjects beyond these was designed, based on pre-existing research and patient and public involvement. Secondly, a reflexive diary was kept in which the researcher described each study interaction (interviews, reading journals, correspondence) and reflected on how these interactions reinforced or challenged the researcher’s assumptions. Analytical ideas were also discussed with the study co-authors (both senior clinical academic nurses with different areas of clinical expertise), to ensure they were coherent to someone without the primary researcher’s assumptions. Finally, a lay summary of the project was provided to study participants and the study patient and public involvement group, inviting them to comment on its accuracy and coherence.

### Patient and Public Involvement

This study is one phase of a larger PhD project, and patient and public involvement has been a feature throughout the entirety of the PhD program. An eleven-person group of people with multiple chronic conditions and carers were convened, and over a period of several weeks they provided feedback on methods and study design, as well as reviewing all participant-facing documentation to ensure it was both coherent and comprehensive. The group also included people with sight, hearing, and cognitive impairments, who were able to advise on methods to ensure that people would not be needlessly excluded due to unsuitable study materials.

### Ethical Approvals and Considerations

The study received a favorable opinion on January 10, 2023, from Scotland A Research Ethics Committee.

The main ethical concern for this study relates to the recruitment of adults with incapacity. As stated above, ethical approval was secured from a committee who have the expertise to review studies which seek to work with this vulnerable population. We still explained the study to both parties, and the legal representative was encouraged to consider what they believed the patient’s wishes to be rather than their own. Provision was made to re-consent participants if they regained capacity during the study; however, due to the chronic nature of the conditions which precipitated their diminished capacity, this did not occur during the study.

To protect participant confidentiality, pseudonyms are used in this report, and any identifiable details have been redacted.

## Findings

This section will begin by describing the sample, before providing a simple description of the work people were engaged in. The way that capacity is distributed will be discussed using Burden of Treatment Theory, before the mediating effect of uncertainty will be explored.

### Participants

Six patient/carer dyads were recruited from the emergency department. All patients had multiple chronic conditions including one of the five index conditions specified. Half of carers were spouses, although adult children, parents, and friends were also represented. Two patients did not have capacity to consent; therefore, consent was obtained through their carer who was also their legal representative. Some carers also had multiple conditions, and some also met the eligibility criteria as patients. While the study adopted a disease-agnostic approach, focussing on the experience of multimorbidity rather than the specific conditions of which it is comprised, it is worth highlighting that of the six patient/carer dyads, five included at least one person who currently or previously had cancer.

Most patients were in their 70s, although the youngest was in their 20s, and while efforts were made to identify and recruit non-white participants, this was not achieved. Earlier research indicates that the study population is approximately 97% white (McParland et al., 2022). Efforts to recruit a socioeconomically diverse sample were more effective. The Scottish Index of Multiple Deprivation (SIMD) is a geographic-area based metric by which multiple forms of deprivation (income, employment, education, health, access to services, crime, and housing) are collated to assign a deprivation rank to every postal-code area in Scotland (The Scottish Government, 2020). Participants in this study ranged from those who lived in the 10% most deprived areas in Scotland to those who lived in the 10% least deprived areas. Slightly more participants were women than men (7:5).

All six dyads took part in the introductory interview, five completed treatment burden journals, and five took part in closing interviews. One dyad completed two separate journals. Most journals were returned in the format of narrative accounts completed on specific dates when participants wanted to share burdensome experiences, although some also sketched pictures alongside these. Only one formal observation session took place, but several ad-hoc observations of medication regimes took place during the interviews. One dyad were lost to follow-up after the introductory interview but had provided consent in advance for their data to be used if they left the study for any reason.

### Simple Description of Patient Work

**Figure 2.**
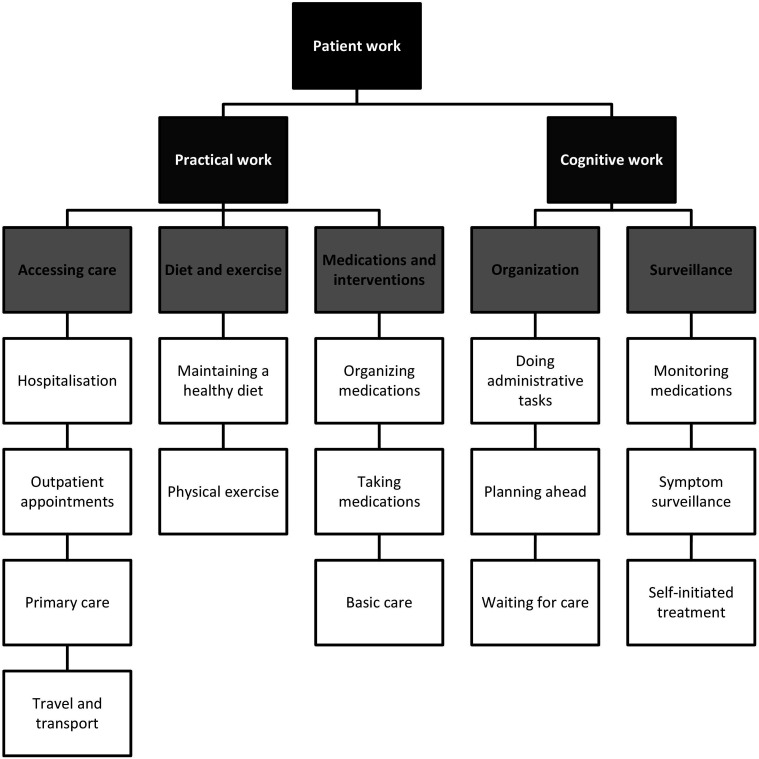
Thematic map of a simple description of patient work.

Participants were engaged in a variety of healthcare tasks which were considered work, and these could be further divided into either practical or cognitive tasks (see Figure 2).

#### Practical Work

The practical tasks undertaken by patients and carers included going to hospital, visiting the general practitioner (GP), and the challenges of travelling for care. Geography, appointment timing, and available transport played a role:

I found it quite stressful with the radiation. You were going first thing every morning for six weeks. First … I mean, your appointment … they give you a block booking. And it was first, 8:30, every morning … (Ruth, carer, interview 1)

There was also the need to maintain good health through diet and exercise, although these were described as small changes rather than adherence to any strict regimen:

Our problem, from my background, is the portion size. We tend to eat too much. So, we have both trimmed that down a bit. And we’re trying to not have as much at lunchtime. Whether that’s good, bad, or indifferent, we prefer having a meal at night. (Irene, carer, interview 2)

Lastly, the day-to-day practical work of taking medicines and other treatments was discussed. One person had been taking almost 80 tablets a day, and several people produced large containers and bags of medicines during interviews. Some struggled with drowsiness, memory-loss, or poor balance, and attributed this to their medications:

I can’t remember back three years ago, you know what I mean, because I was on a lot of drug substitutes and all the painkillers and all that I was on. I used to be on pregabalin as well, do you know what I mean, it was just like … It was horrendous. I was on the methadone. I was on about 80 milligram of Valium a day… (Mel, patient, interview 1)

Medications made up most of the self-administered interventions, but it is also important to highlight that tasks such as stoma care, self-catheterization, and enteral feeding were among the practical tasks undertaken by patients and carers.

#### Cognitive Work

Cognitive work is characterized by the passive yet ongoing work of organization and surveillance. The day-to-day administration and forward planning required was exemplified in several cases, such as Andy who was overseeing adaptions being made to his mother’s home, or Ruth who had been applying for financial support for adaptions to her and her husband Tom’s home:

I had a carry on … nine calls before I got through! Nine … kept cutting me off, cutting me off, put you through to this department, cut you off. It was a carry on. (Ruth, carer, interview 2)

Despite efforts to organize and coordinate care, there were often long and indeterminate waiting periods. This waiting was divisive, eliciting emotional responses in some while being met with equanimity by others:

We waited an hour and a half and when she came up in the ambulance, she should have been taken right away rather than wait all that time and other people getting taken who came in after my mum were getting taken. And that’s why I jumped up and I went and said “listen, hold on …” (Andy, carer, interview 1)

Surveillance was associated with three inter-related concepts: symptom surveillance, monitoring medication, and self-initiated treatment. Symptoms may emerge independently or in response to medication changes, and medication changes may be the necessary self-initiated treatment in response to such symptoms. For example, Ruth was on multiple medications including Bendroflumethiazide, which her doctor increased due to her high blood pressure (symptom surveillance). She then developed a cough (symptom surveillance) and treated this with cough-medicine (self-initiated treatment). When this failed to resolve, she considered the possible link with the Bendroflumethiazide (monitoring medication) and with her doctor’s agreement, stopped the medication:

I was getting a cough all the time. I bought three different cough [medicines] and it was still here and I went … and then they upped the dose on it ‘cause my blood pressure was still high. They upped the dose on [the Bendroflumethiazide] and I coughed even worse and then I realized … I went, that’s that pill, and I read the effects. So, I stopped that on Friday. (Ruth, carer, interview 1)

### Workload and Capacity

The preceding section provides a simple description of the type of work undertaken by patients and carers. At a fundamental level, Burden of Treatment Theory is concerned with the balance between workload and capacity; this section describes how these concepts relate to people with advanced multimorbidity and their carers.

#### Agents and Systems

Individual patients have a general potential to undertake the work of patienthood (agency), and intrinsic factors such as the physical and mental health of an individual contribute toward this. However, the ability to express this agency is further extended or constrained by locally available opportunities and the way that health systems are organized. For example, some patients could see their GP easily, while others had a long wait for an appointment. In another example, the health board in which the research was conducted were trialing a hospital-at-home service, and some participants were very positive about it, highlighting how it had prevented admission to hospital:

I have that hospital at home service, so that way my mum can get treated at home the same way that she got treated in hospital. Which was better, because that way she was in her home, she had people round about her all the time and so it made more sense. (Andy, carer, interview 1)

However, the eligibility criteria limited access to older adults, even when younger people could benefit from it. Stephanie’s young adult son Nick had multiple complex needs, was unable to mobilize, and had several emergency admissions, yet was unable to receive the hospital-at-home service. When asked if she thought the service could have avoided previous admissions, Stephanie responded:

I think so, because I mean, it’s a brilliant service, and they really offer everything that can be provided that Nick would require in hospital, such as oxygen therapy … he’s got a home nebulizer anyway that we need if … we can use if needed, but intravenous, they can provide intravenous antibiotics. (Stephanie, carer, interview 2)

The agency of both patients in these examples was limited by their physical health, and both relied on family carers. However, the unequal distribution of opportunities meant that Nick and Stephanie’s workload increased by having to attend the hospital, while Andy’s mother remained at home.

#### Relationality and Resources

The interactions between individual patients (agents) and healthcare services (systems) are mediated by several other factors. Of relevance is the relational network of the patient, which includes their carer and also contacts within the healthcare services. We categorized network members as either primary carers (e.g., spouses and family members), secondary carers (including the extended family and friends/neighbors), and healthcare contacts who could be considered part of the network (such as well-known GPs or specialists). Due to the eligibility criteria of this study, everyone had a primary carer, and there were examples of how they extended the capacity of individual agents. Often this was one-directional, such as in the case of Stephanie and Nick or Andy and his mum Patricia in the previous section, yet sometimes the differentiation between patients and carers was less clear. In the case of husband and wife Tom and Ruth, each could be said to reciprocally care for the other, driving each other to appointments, or organizing one another’s medications. For spouses Mel and Scott, there was also an element of reciprocity, although due to her poorer general health (and therefore diminished agency), Mel relied to a greater extent on Scott to extend her capacity to undertake work. This encompasses not only the practical day-to-day tasks but also emotional and cognitive work:

I used to always say to Scott, years ago, Scott, see if I ever have a heart attack or I’m having one or a stroke or they tell me I’ve got cancer or I’m dying or something like that, you make sure they tell you first, Scott. Then you tell me because I wouldn’t be able to handle getting told. (Mel, patient, interview 1)

The secondary tier of carers was also important. Even when they were infrequently accessed, simply knowing support was available if needed seemed to provide reassurance. Barbara’s adult children had long moved far away, and her primary carer was her friend and neighbor Theresa; however, when questioned about whether her children would come visit if she was unwell, her response was unequivocal:

Yes! Oh, they would be on the first plane. (Barbara, patient, interview 2)

Tom and Ruth also had an example of this relational support, when describing Tom’s recent fall at home:

Once I got him in bed, I thought, he needs the hospital. And I can’t get him out of bed. Well, I just phoned and (Tom’s son and daughter) were down there within half an hour. (Ruth, carer, interview 1)

The relational network may also include healthcare staff when such individuals are well-known to the patient and their carers. One example was Bill’s oncologist, who would visit him on the ward to check-in, even when he was admitted to a different hospital for non-cancer related reasons:

… she’s a bit like an elf, she pops up unexpectedly. I was in, was it, I can’t remember (which hospital) the first time, and she just appeared. She said, I was in the building, and I thought I’d come and see you, and we’ll talk about this (admission). (Bill, patient, interview 1)

Access to material and informational resources is also central. Healthcare in the United Kingdom is universal and funded by the taxpayer, yet financial resources can still mediate treatment burden by making it easier to travel for care (getting taxis or paying parking fees), enlisting paid help with daily activities, being able to buy equipment and over-the-counter medicines, or by enabling people to access private healthcare (avoiding NHS waiting lists):

I had to pay £250 just to see a neurologist. But do you know what, it was worth every penny because he said I’ve got a (specific condition). So that’s me diagnosed within 20 minutes. (Andy, carer, interview 1)

Material resources may also involve equipment which helps patients and carers manage burdensome treatment. Several discussed home adaptions and anticipated mobility aides, yet the way in which these mediate burden is most striking when something which has become a normalized part of a routine is removed. An example of this was when Nick was admitted to hospital, and a problem with basic mobility equipment (which Nick used at home) meant that his parents had to manually reposition him themselves:

… there was just the bit of frustration about the hoist, there being three hoists available that we couldn’t use, because they … apparently, they weren’t working or … I’m not sure, there’s the whole reason. (Stephanie, carer, interview 2)

### Uncertainty and Burden

**Figure 3.**
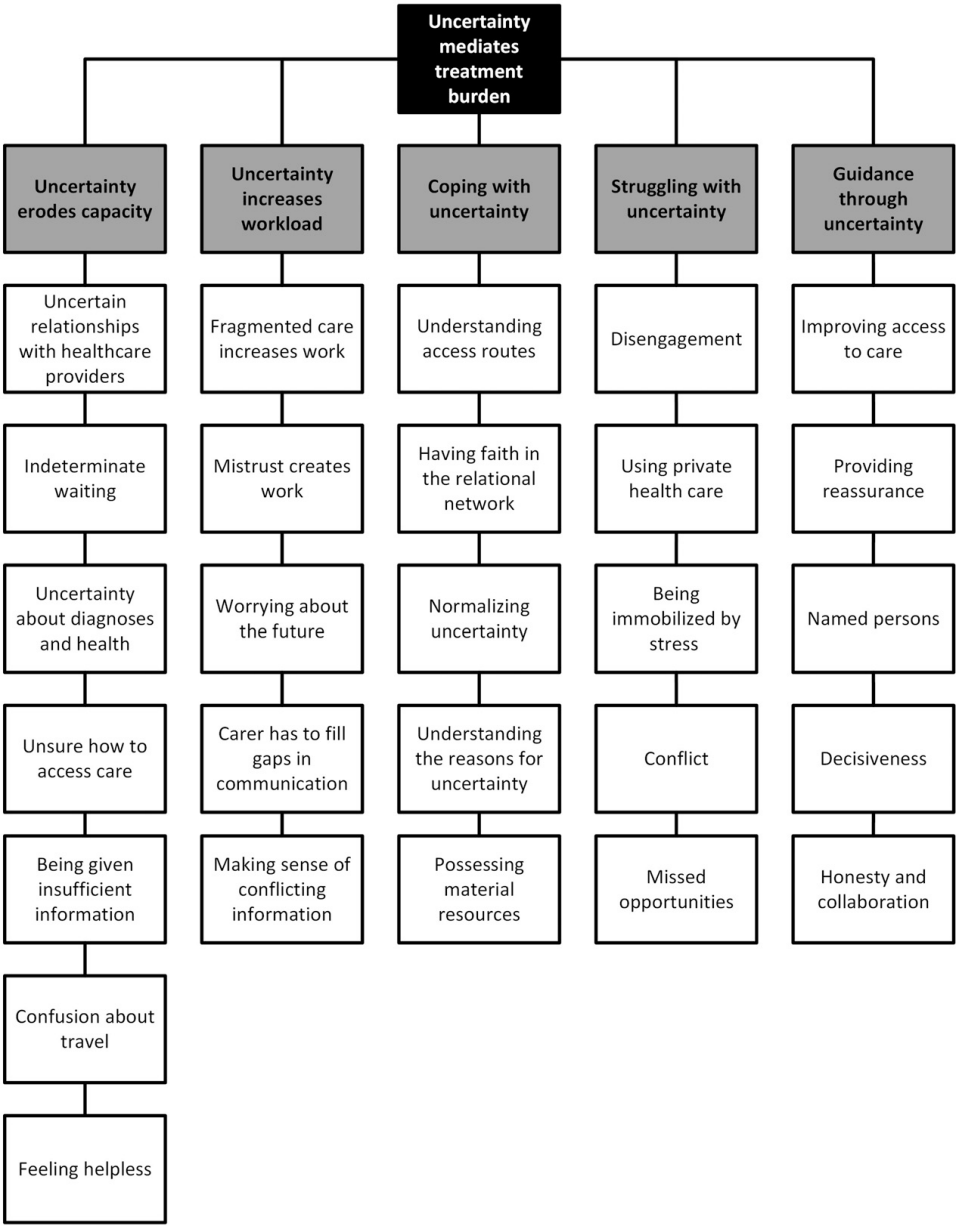
A thematic map of the relationship between uncertainty and treatment burden.

Five themes were constructed to describe the relationship between uncertainty and treatment burden. The first two themes deal with the interaction between these concepts, specifically the way that *uncertainty erodes capacity* and how *uncertainty increases workload.* Two further themes provide evidence of these interactions, describing how some people are *living with uncertainty* while others are *struggling with uncertainty.* Finally, a means of improving care is suggested, namely, providing *guidance through uncertainty* (see Figure 3).

#### Uncertainty Erodes Capacity

Uncertain relationships with healthcare providers are a form of social uncertainty, in which a lack of trust means people are unable to build and maintain relationships with healthcare providers. Ruth gave the example of feeling unable to make herself understood when her doctor spoke to her in what she perceived to be a patronizing manner:

I think when you’re over 70, you’re treated like a geriatric. They start speaking slowly to you. They start speaking louder to you, as though you don’t understand. I might be 73, but I’m still compos mentis. (Ruth, carer, interview 1)

Waiting was also a significant source of uncertainty, and one which eroded capacity through its indeterminate nature. Travelling to and from hospital was a frequent source of such waiting, as noted by Theresa while waiting for her friend Barbara to be seen in the emergency department:

We weren’t sure if they were going to keep you in, that was the thing …. and [our other friend] was waiting to find this out, and she had to wait the whole day, which was the same as me the last time. To wait the whole day to know that she was going to be staying in. Whereas if they had said she is going to be staying in that would have been fine, you know, but it takes a long time to do that. (Theresa, carer, interview 2)

Whether waiting for an outpatient appointment for several months or waiting a few hours for transport home from hospital, indeterminate waiting requires the person to maintain a state of vigilance, simultaneously poised to act yet unable to do so. Theresa, for example, felt unable to leave Barbara’s side to make arrangements for her medication delivery, to pick up food, or to plan for her own travel home until she had the confirmation that Barbara would be admitted to hospital.

Uncertainty around diagnoses and changing states of health was also common, the conscious ignorance characterized by some as stepping into “the unknown”:

On the initial visits to the hospital, every step of this is a learning curve for us, because we’re going into the unknown all the time. (Irene, carer, interview 1)

This was further complicated by difficulties in accessing care, with uncertainty around access limiting the ability of some to get the support they needed:

So, I might need the (carer’s support service), or the assistance they give. And I don’t even know what’s available up there, I’ve no idea. And who gives you that information … where do you access that? (Irene, carer, interview 1)

Information flow also leads to uncertainty. Mel’s frustrations at not having her hospital discharge medications explained leading to problems with her ongoing medication management is one example:

When I’m getting discharged from hospital, I always say to them, can I check the list of what you’re putting through for the pharmacy? They always say, no, we know what you’re getting. We’ll give you the right things but they never ever let me check that list. I don’t know why because they should do because it’s my medicines. (Mel, patient, interview 1)

Insufficient information flow limits capacity through a simple mechanism: people cannot enact work which they lack the information required to conceptualize.

Confusion about travel was another feature, particularly with ambulances or patient transport services, and this often led to increased workload. After a long wait for an emergency ambulance, Andy ended up cancelling the request because his mother Patricia didn’t feel well enough to go to the hospital late at night; however, this only led to further waiting the following day:

It took me until the Tuesday until I got my mum to the hospital because of the waiting time for the ambulance. I had to cancel it at 9:30 at night after waiting all afternoon because they said that it wouldn’t be there until 11 o’clock at night and the stress could have killed her. (Andy, carer, journal)

The culmination of these mediating factors often led to a feeling of being overwhelmed, which further eroded the capacity to undertake work, as described by Irene, when comparing her own feelings to those of her husband Bill:

Bill is coping, I think, very, very well with this. Because he’s not a worrier, and he says, there’s no point in worrying because I can’t really do anything about it, everything that’s being done is being done. I mean, (Bill’s oncologist) has left no stone unturned, she’s been really excellent. And he has a great trust and rapport with her. Obviously, my situation is different, I’m onlooking. So, I feel out of control. (Irene, carer, interview 1)

#### Uncertainty Increases Workload

Uncertainty also increases the perceived workload of patients and relational network members. Navigating fragmented care or siloed working practices means people had to repeat stories and relay information across multiple providers, as Scott described when talking about his wife Mel’s experience with her GP:

If you go to see a doctor, “right, (Mel), what can I do for you today?” She’ll start to tell him. “Oh, I can only deal with one thing.” Well, she’s very complex, you know, so she’ll be saying, well, my legs are swollen and I’ve got these little spots, stuff like that, but “I can only deal with one thing.” (Scott, carer, interview 1)

The way in which mistrust and uncertainty limit capacity has already been discussed, but it can also increase work. Not trusting healthcare providers led to people doing their own research into treatments, taking on tasks (such as organizing medications) which they thought would be done incorrectly, or feeling obliged to present themselves in certain ways to appear credible:

I’ve got a friend that always gets all her makeup on and gets all dressed up and goes because … ’cause of her age, because she says, they (healthcare professionals) think you’re old and decrepit and they don’t bother. (Ruth, carer, interview 1)

Uncertainty about the future is also a common feature of chronic, palliative, and multimorbid conditions, yet worrying about what may happen also constitutes a form of patient work, one which is emotionally and cognitively demanding:

I’ve been quite weepy, easily moved to tears, and anxious, frightened about what’s ahead of us. Which is unknown because we don’t know how this is going to progress. Do we run out of options for treatments, what happens then? (Irene, carer, interview 2)

Another form of work borne of uncertainty relates to poor communication, and where uncertainty leads to carers having to fill the gaps in what is communicated between healthcare providers and carers. Andy found himself often in this position, liaising between his mother and her clinicians about medications:

My mum was adamant that she wanted to take them at the beginning, but I didn’t want her to take them, but I couldn’t stop her. Because obviously I said to her, those two tablets together, they’re not really a good combination. (Andy, carer, interview 1)

Perhaps most challenging of all is when information is not simply inadequate or poorly communicated, but when it is in conflict with other information. Bill had taken to walking circuits near his home in order to lose weight and improve his breathing (as recommended by his primary care team), but at the same time his wife Irene worried that this was at odds with what his cancer consultant wanted:

The only thing that worries me slightly is the oncologist said she really didn’t want him to lose too much weight. I think she wants him to have a backup. (Irene, carer, interview 2)

In these cases, the work of reconciling conflicting information often falls to patients and carers.

#### Living With Uncertainty

Stephanie (who worked in healthcare) used her knowledge of current system pressures to contextualize some of the uncertainties she faced when her son Nick was in hospital:

… I understand the nursing staff, the medical staff and porters and the cleaning staff are absolutely fantastic and (my husband) and I can’t praise them enough. But everyone is so stretched, you know, in hospitals, and I completely appreciate that it must be so frustrating for staff working there ’cause it’s so short staffed. (Stephanie, carer, interview 1)

People who demonstrated an ability to cope with uncertainty often had a better understanding of the access routes to receiving care and of the underlying reasons for uncertainty.

The earlier case of Tom and Ruth is an example of how material and relational resources can help people cope with uncertainty. They felt comfortable managing their healthcare workload and also their broader social life (including a lot of foreign travel), in the knowledge that their family were always available to help them out. Bill and Irene similarly felt secure in the support of friends and neighbors, and also reflected that they were very fortunate in having a financial buffer to protect them from future uncertainties:

We can afford, fortunately, to get more help if we require it. (Irene, carer, interview 2)

Material and relational resources (and an awareness of how readily these could be accessed) were strongly linked to the ability of people to cope with uncertainty. This often resulted in a normalization of uncertainty, or an acceptance which limited the negative effects of living with uncertainty.

#### Struggling With Uncertainty

There were also cases where the burden of uncertainty was seen to be overwhelming, which often lead to further worsening of relationships and ultimately more uncertainty. Mel reported being engaged in legal action against her primary care provider, stemming from uncertainties around why she couldn’t access changes to her medications. This conflict then led to disengagement, with Mel limiting her contact with the health center, and being unable to switch to another provider due to geographic restrictions:

I mean I’m suing my doctor at the moment. I’ve just changed my doctors as well there to a different one in the health centre because I tried to get rid of the health centre completely but that’s the only health centre that will take on this postcode, do you know what I mean, so I’ve got to go with a surgery in that health centre … I didn’t want to be with that health centre full stop. (Mel, patient, interview 1)

In the same way that mistrust had the potential to increase work, uncertainty also leads to conflict and disengagement. Occasionally this disengagement would result in people paying for private healthcare. All people in the study were UK citizens and access to primary and secondary care through the NHS was available to everyone for all procedures discussed in the study. This type of disengagement merits attention as it represents a system where those with financial means have greater capacity to act than those without. However, accessing private healthcare was not solely described by people who also reported financial security.

Overwhelming uncertainty also led to missed opportunities or delayed access to services. Irene, as discussed earlier, was struggling with her husband Bill’s cancer diagnosis, but due to multidimensional uncertainties (including transport to the center, her eligibility to access the service, worries about Bill’s health, plus initially feeling let down by center staff when she first tried to get help), she had felt unable to access support. By the end of the study, Irene managed to speak to someone, but by this time she had been struggling with these uncertainties for far longer than she could have been. Once these were overcome, she recounted feeling relieved:

So, she took me to a wee area, and we sat and chatted, and I told her what was happening. And I said, you know, it’s a year, and she said, and you’re only finding us, and I said, yes. And she said, God, that’s awful. Partly maybe my own fault for not looking more at the leaflets and things on the ground floor. (Irene, carer, interview 2)

#### Guidance Through Uncertainty

After several challenging admissions during which Patricia and her son Andy were adamant that she wouldn’t be admitted to the hospital again, but Patricia was quick to recount how one ward and the nurse caring for her there had been different:

I told that staff nurse, I said, “I’ll never come back,” but I said, I tell you now, if I had to come into the hospital, as long as I know they’re putting me into your ward, I’ll come in. I would come in, I wouldn’t have any problem. (Patricia, patient, interview 2)

There were several instances of people who acted as guides through uncertainty, and they were often characterized by their ability to reassure, to make decisions, to be honest and collaborative in how they planned care. Crucially, these were often *named* individuals who offered continuity in an otherwise uncertain landscape. The person Irene met at the center did something which is central to coping with uncertainty: they offered reassurance that the service was *for* her, that she could access it whenever she needed support. Ruth provided an example of turning to a trusted respiratory nurse when she couldn’t get an appointment for a (non-respiratory) issue, while Bill’s relationship with his oncologist was a constant source of reassurance.

## Discussion

The treatment burden experience of people with advanced multimorbidity is characterized by a combination of practical tasks (such as accessing care, maintaining diet and exercise, or managing medications), and the cognitive work of organizing and self-surveillance. Burden of Treatment Theory provides an effective lens through which treatment burden in advanced multimorbidity can be understood; agents interacting with a system which shapes and constrains their capacity to undertake patient work, while these agents leverage relational and material resources in order to extend their capacity. Uncertainty plays a major role too, however, and it is this conscious awareness of ignorance (about health, about the services available, about the healthcare professionals they interact with, and about the future) which has the potential to destabilize the delicate balance of workload and capacity described by Burden of Treatment Theory. In order to restore this balance, there is a need for people who can offer continuity and guidance through uncertainty.

The types of tasks undertaken in this study echo what has been described in a range of other studies, in other populations including people with multimorbidity in Malawi (Chikumbu et al., 2022), people with cancer (Adam et al., 2023), and people who have had a stroke (Gallacher et al., 2013). Recent research has shown a relationship between higher levels of self-reported treatment burden (measured using validated tools) and lower health-related quality of life (Gebreyohannes et al., 2023). This reflects the experience of several participants who were struggling with their treatment regimens due to burden and uncertainty. The types of uncertainty experienced mapped effectively onto those described by Etkind et al. (2022); given that these were based on a systematic review of studies involving people with advanced multimorbidity, this provides reassurance that the themes discussed in our study may have wider relevance. Extending Burden of Treatment Theory in order to better describe the experience of different populations has also been done elsewhere; for example, in one study in South Africa (van Pinxteren et al., 2023), the concept of “persistent precarity” was described as mediating the extent to which people could cope with their workload, in a similar way to how uncertainty affected the population of our study. There are additionally tools designed to handle uncertainty in people with advanced multimorbidity, although these are concerned primarily with clinical uncertainty (Ellis-Smith et al., 2021) rather the multidimensional uncertainty described by Etkind et al. (2022).

**Figure 4.**
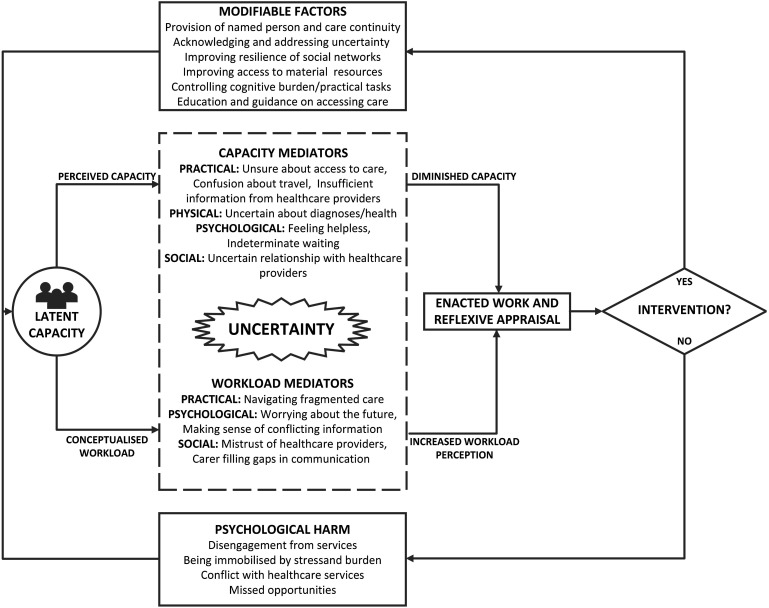
A conceptual model of the relationship between workload, capacity, and uncertainty.

Where this study makes a unique contribution is in linking the concepts of workload, capacity, and uncertainty, in a conceptual model which draws on the findings of the study alongside Burden of Treatment Theory and the concept of Total Uncertainty and Uncertainty Tolerance (Figure 4). The model proposes that the latent capacity of patients and carers to undertake work is mediated by practical uncertainties (such as poor information and confusion around access or travel), physical uncertainties (about the individual’s health), psychological uncertainties (feeling helpless, often in the face of indeterminate waiting for care), and social uncertainties (feeling uncertain about healthcare providers). Meanwhile, the conceptualized workload is mediated by other practical uncertainties (navigating fragmented care), psychological uncertainties (worrying about the future or making sense of conflicting information), and social uncertainties (mistrusting healthcare providers or requiring caregivers to relay or interpret information). With diminished capacity and a perceived increase to workload, the ability to enact and reflexively appraise the work is limited, which can lead to disengagement, conflict, missed opportunities, and being immobilized by stress and burden.

However, drawing on the examples within this study and the work of May et al. (2014), we propose that there is scope to intervene and break the cycle of diminished capacity and increased workload. The value of provider continuity is a long-established concept in healthcare, and one which has been shown to yield myriad benefits including reduced mortality (Baker et al., 2020) and better patient satisfaction (Van Walraven et al., 2010). Continuity of care may extend the agency of patients and carers, but providing this relationality requires structural changes to the way health services are organized.

While this paper argues that multidimensional uncertainty can diminish the ability of people to cope with the work of patienthood, it is important to also acknowledge that (as with treatment burden) the goal should be to achieve satisfactory balance when dealing with uncertainty. As Etkind and Koffman (2016) suggest, uncertainty is not necessarily something which can or should be “removed” by the clinical team. For example, it may play a protective role in coming to terms with life-limiting illness, and addressing this uncertainty may be more closely aligned with the goals of clinicians than patients and carers. We argue that achieving this balance is fundamental in helping people with advanced multimorbidity navigate complex systems, and to begin to acknowledge, address, and live with the multidimensional causes of uncertainty.

Mason (2022) suggests that both certainty and uncertainty can be considered either safe or unsafe from a therapeutic perspective. Using Mason’s framework, we can understand the fallibility of striving for safe certainty when caring for people with advanced multimorbidity, as it is unrealistic for clinicians to expect those in their care to both fully comprehend and accept the myriad uncertainties they experience. It may be more realistic to create a context of safe uncertainty, characterized as a dynamic state in which explanations for uncertainty are constantly evolving and co-created through the therapeutic relationship.

The relational network and material resources also play a major role in navigating burden and uncertainty, so any intervention should proactively seek to ensure the resilience of such networks, and to facilitate access to community resources. Strong social support and social activity help protect cognitive function (Kelly et al., 2017), which is particularly important given the need to both educate people about the ways of accessing healthcare effectively while also seeking to control the cognitive burden placed on them by healthcare tasks. Returning to our epistemological assumptions, we allow for multiple models offering different perspectives on the same phenomena; therefore, this conceptual model should be appraised based on the extent to which it is useful in practice and developed accordingly.

### Strengths and Limitations

This study involved in-depth serial interviews with people with various combinations of conditions and included the important voice of caregivers. We adopted flexible methods to encourage participants to engage with the study team in different ways, including participant-led journaling. The study was designed with support from a large patient and public involvement group, and findings were reported back to both study participants and the members of the patient and public involvement group. It is also the first study to our knowledge which explicitly links the concepts of treatment burden and uncertainty, and we have produced a conceptual model which others may use to explore whether similar relationships are to be found in other populations.

The voice of healthcare providers is absent; however, we believe that focussing only on the patient and carer perspective was warranted in this instance. Future research should also explore how healthcare providers understand treatment burden and uncertainty. We also note that while we encouraged participants to take part in observation sessions, only one such formal session was conducted. However, most interviews were conducted in individual’s homes, which meant that more informal observations (such as seeing drug storage and organization methods, or how people coped with mobility aids in their home) were possible. We have also focussed on the phenomena of treatment burden and uncertainty from the perspective of patient/carer dyads, and the distribution of burden across relational networks. Interviewing participant dyads together and the adoption of this analytical approach limits the extent to which we can draw conclusions about patients and carers as discrete agents within this network.

## Conclusion

People with advanced multimorbidity and those who care for them are navigating a complex healthcare system while trying to balance the work they are expected to do with their capacity to undertake this work. Uncertainty, in its many forms, destabilizes these efforts. We propose a model of uncertainty and burden which can be used to understand these relationships, and we recommend changes to how care is provided, particularly through better continuity.

## Supplemental Material

Supplemental Material - Treatment Burden and Uncertainty in the Context of Advanced Multimorbidity: A Focussed Ethnography Supplemental Material for Treatment Burden and Uncertainty in the Context of Advanced Multimorbidity: A Focussed Ethnography by Chris McParland, Bridget Johnston, and Mark Cooper in Qualitative Health Research
