# Supplementary material for: Treatment Burden and Uncertainty in the Context of Advanced Multimorbidity: A Focussed Ethnography
Source: Qual Health Res. 2025 Mar 14;36(1):107–23. doi: 10.1177/10497323251320836 (PMC12675835; doi:10.1177/10497323251320836)
Supplement: Supplemental Material - Treatment Burden and Uncertainty in the Context of Advanced Multimorbidity: A Focussed Ethnography [file sj-pdf-1-qhr-10.1177_10497323251320836.pdf]

A focussed ethnography of multimorbidity, treatment burden and burden for carers using reflexive qualitative methods (EMBARQUE study)

Interview topic guide

The following topic guide can be used to ensure relevant areas are covered across the series of interviews. **They should not be covered in one study visit,** and some participants may be more focussed/interested in some areas than others. The questions have been developed based on two validated questionnaires designed to assess treatment burden in chronic illness[1] and in multimorbidity[2]. Prompts are *italicised* – these do not need to be asked directly but may be used to elicit further information if necessary.

Questions can be phrased differently dependant on whether they are being directed to the patient or their carer – see comments in **[square brackets].**

Topic 1 - Medicines

1. Can you tell me about the medications **[you/your friend or relative]** take?

    Number of medications?

    Types of medications? (pills, liquids, injections, inhalers, topical)

    Frequency of medications?

    Injections – bruising, bleeding, scars?

    Pills – hard to swallow, taste bad?

2. How do **[you/your friend or relative]** remember to take the right medication at the right time?

    Think about alarms, keeping notes getting others to remind them.

    Missed doses or incorrect doses taken by accident?

3. Are there any special rules **[you/your friend or relative]** need to obey when taking medicines?

    Need to eat/fast?

    Need to lie down, get undressed to apply topical drugs?

4. Do **[you/your friend or relative]** collect **[your/their]** own medicines/prescriptions?

    If not, do ***[you/they]*** *arrange someone else to do it? Pay someone else to do it?*

    If not, do ***[you/they]*** *have to make phone calls, go online etc to organise?*

Topic 2 – Self-management activities

5. Do **[you/your friend or relative]** have to do anything to monitor **[your/their]** conditions?

Blood sugar? Blood pressure? SpO2?

Symptoms?

*If any equipment is used – how do **[you/they]** maintain it?*

6. Have **[you/your friend or relative]** had to make any changes to the way **[you/they]** eat or exercise because of health conditions?

Had to lose weight or restrict certain foods?

Had to gain weight or include certain foods?

Had to increase exercise or undertake certain exercises, including physical therapy?

Had to abstain from types of exercise which were previously undertaken?

7. How do **[you/your friend or relative]** keep informed about the chronic conditions **[you/they]** have?

Any formal patient education?

Education delivered by informal carers?

Self-directed research and education?

Topic 3 - Accessing health care

8. What health professionals do **[you/your friend or relative]** see for **[your/their]** conditions?

What <u>specialists in particular</u>, and how often?

Are they in different places?

Are they all on different days?

9. How do **[you/your friend or relative]** get to appointments?

Driving or getting public transport?

Are they far away or far apart if on the same day?

Getting informal or NHS transport?

10. Do appointments take a long time?

Long wait times in clinics?

Gaps between appointments if on same day?

Waiting for transport?

11. How do **[you/your friend or relative]** arrange and keep track of appointments?

*Do **[you/they]** arrange your [their] own appointments or are they sent to you [them]?*

*Do **[you/they]** have to get someone else to help arrange appointments?*

*Do **[you/they]** get reminders of appointments?*

*Do **[you/they]** have to keep a diary?*

*Do **[you/they]** have to get time off work or other commitments?*

12. Do **[you/your friend or relative]** have to arrange any other types of care or support?

Paid or informal carers?

District nurses?

Community support?

13. Do **[you/your friend or relative]** ever get conflicts with appointments being booked together?

Appointments booked on the same day?

*How do **[you/they]** resolve these conflicts?*

14. Do **[you/your friend or relative]** ever have to access emergency or out-of-hours care?

Time spent, travelling, arranging carers as above

15. Do **[you/your friend or relative] have** to pay for anything that helps **[you/them]** manage **[your/their]** conditions?

Non-prescription medications?

Adaptions to home environment?

Carers (including informal)?

Travel?

Loss of work?

16. Is there any paperwork or administrative work **[you/your friend or relative]** have to do because of **[your/their]** conditions?

Applications for support related to treatment

Anything related to employment

Reimbursement for costs incurred (travel etc).

Topic 4 – Social and personal

17. Can you tell me about any help **[you/your friend or relative]** get from family or friends to manage **[your/their]** conditions?

Recap anything which has been mentioned up till now

*Do **[you/they]** have to organise and manage this support?*

18. Has **[your/your friend or relative's]** healthcare had any impact on **[your/their]** social relationships?

This may be positive or negative

Don't just focus on immediate caregivers – has it affected friendships or work relationships?

19. Does the healthcare **[you/your friend or relative]** use have any impact on the way **[you/they]** feel about [**yourself/themselves]**?

Use example of 'frequent healthcare reminds me of my health problems' – a theme identified by UK participants in the development of the treatment burden questionnaire

Topic 5 – COVID-specific impact

20. Did the way **[you/your friend or relative]** access healthcare change during the coronavirus pandemic?

Remote appointments?

Fewer appointments?

Same contact with carers and community services?

21. Were there any positive aspects to these changes?

*Did these reduce treatment burden in any way, and is there anything which **[you/they]** would like to continue if given the choice?*

References

1. Tran V-T, Harrington M, Montori VM, et al. Adaptation and validation of the Treatment Burden Questionnaire (TBQ) in English using an internet platform. *BMC Medicine* 2014;12(1):109. doi: 10.1186/1741-7015-12-109

2. Duncan P, Murphy M, Man MS, et al. Development and validation of the Multimorbidity Treatment Burden Questionnaire (MTBQ). *BMJ Open* 2018;8(4):e019413. doi: 10.1136/bmjopen-2017-019413 [published Online First: 2018/04/15]
